# Effective gossypol removal from cottonseed meal through optimized solid-state fermentation by *Bacillus coagulans*

**DOI:** 10.1186/s12934-022-01976-1

**Published:** 2022-12-01

**Authors:** Zhenting Zhang, Danlu Yang, Ling Liu, Zhangbing Chang, Nan Peng

**Affiliations:** 1 State Key Laboratory of Agricultural Microbiology, Hubei Hongshan Laboratory, College of Life Science and Technology, Huazhong Agricultural University, Wuhan, 430070 Hubei People’s Republic of China; 2 School of Public Health, Guizhou Medical University, Guiyang, 550025 Guizhou People’s Republic of China; 3 National Engineering Research Center of Microbial Pesticides, Wuhan, 430070 Hubei People’s Republic of China

**Keywords:** Cottonseed meal, Free gossypol, *Bacillus coagulans*, Detoxification, Solid-state fermentation

## Abstract

**Background:**

Cottonseed meal (CSM) is the main by-product of the cottonseed oil extraction process with high protein content, which is an important protein source for feed industry. However, CSM contains free gossypol (FG), a toxic substance that is detrimental to animal health and greatly limits its application. Microbial fermentation is currently considered to be one of the most effective methods to reduce FG and other anti-nutritional factors in CSM. Previously, yeast and bacteria species are used for degradation of FG in CSM, but showing less detoxification efficiency. *Bacillus coagulans* combines the properties of both lactic acid bacteria and *Bacillus*, producing both lactic acid and spores, and is considered a potential probiotic. In this study, we aimed to evaluate and optimize the effect of the solid-state fermentation process using a *Bacillus coagulans* to gossypol removal contained cottonseed meal.

**Results:**

36 *B. coagulans* strains were isolated and found to have the ability to remove free gossypol. Through the evaluation of strains and optimization of fermentation conditions including fermentation temperature, ratio of material to water, inoculation amount, fermentation time and pH, we have established a solid-state fermentation process using a *Bacillus coagulans* strain S17 on CSM substrate with 1:1 of the material-to-water ratio, 15% (v/w) seed inoculation, 2% expanded corn flour, 1% bran, and 0.3%-0.8% metal irons at 40 °C for 52 h. After fermentation, the FG content in CSM was reduced from 923.80 to 167.90 mg/kg with 81.83% detoxification efficiency. Meanwhile, the crude protein content in CSM increased from 47.98 to 52.82%, and importantly, the spore concentration of strain S17 reached 1.68 × 10^10^ CFU/g dry material.

**Conclusion:**

The study showed that *B. coagulans* have the potential strong ability to degrade free gossypol through cottonseed meal fermentation. This study presents a feasible process for improving the resource utilization rate and nutritional value of CSM via solid-state fermentation through *B. coagulans* S17.

**Supplementary Information:**

The online version contains supplementary material available at 10.1186/s12934-022-01976-1.

## Background

Cottonseed meal (CSM) is an important plant-derived protein in animal breeding industry [1]. However, CSM contains a high-level of free gossypol (FG), a naturally occurring animal toxin that limits the use of CSM and its derivatives in animal feed [2]. FG affects the function of several enzymes, alters the properties of cell membranes [3], and interferes with the use of mineral elements [4]. FG also affects males and females fertility in ruminants and monogastric animals [5, 6]. In addition to being toxic to animals, gossypol residues can be transferred to meat, milk and eggs [7–10], which is harmful for human healthy.

Many methods have been developed for degradation of FG in CSM. The use of organic solvents, such as acetone and ethanol, can extract FG from the CSM, therefore reducing FG content in CSM [11]. However, extraction method requires a large amount of water, loses protein content and reduces the protein quality [9]. Another option is to promote the binding of FG with other compounds, such as FeSO_4_ or Ca(OH)_2_ [12]. However, treatment with FeSO_4_ can cause discoloration of animal feed, reduce palatability and other nutrients in CSM [13].

Microbial fermentation is currently considered to be one of the most effective methods to reduce FG and other anti-nutritional factors in CSM [14]. In addition, the metabolic activities of microorganisms in the fermentation process may produce enzymes, vitamins and some unknown compounds into CSM [15]. All of these have a significant impact on the growth and health of animals, expanding the application of CSM in animal husbandry. Yeast species, such as *Saccharomyces cerevisiae*, are the most used microorganisms for CSM fermentation [14]. However, in an aerobic environment, *S. cerevisiae* produces alcohol rather than cell biomass [16], limiting the animal’s access to nutritious yeast biomass, such as proteins, important amino acids and vitamins. *Bacillus* can degrade FG, but some *Bacillus* species decarboxylate or deaminate in amino acid metabolism, produce pungent ammonia, affect the flavor of fermentation [15]. On the other hand, lactic acid bacteria (LAB) can also reduce the contents of anti-nutritional elements in CSM and produce a large number of volatile substances, such as lactic acid and acetic acid during the fermentation process, which can improve the palatability of feed [17]. However, the ability of LAB to secrete enzymes is weak, resulting in less improvement of the nutritional value of CSM [18]. *Bacillus coagulans*, a lactic acid producing, spore-forming bacterial species [19], has been recognized as an effective probiotic [20, 21]. The optimal growth temperature for *B. coagulans* is 50 °C [22], which shows advantages for lactic acid production [23, 24] and inhibiting bacteria contamination during fermentation [25, 26].

In this study, we aimed to evaluate the effect of *Bacillus coagulans* on gossypol removal while promoting the nutritional value and utilization rate of cottonseed meal. We isolated and characterized 36 *B. coagulans* strains, and established a solid-state fermentation process for degradation of FG in CSM using one of these strains with the best fermentation performance, which greatly improved the utilization rate of cottonseed meal and improved the application of cottonseed meal in animal breeding.

## Results

### Isolation of 36 *B. coagulans* strains from fecal samples

**Fig. 1:**
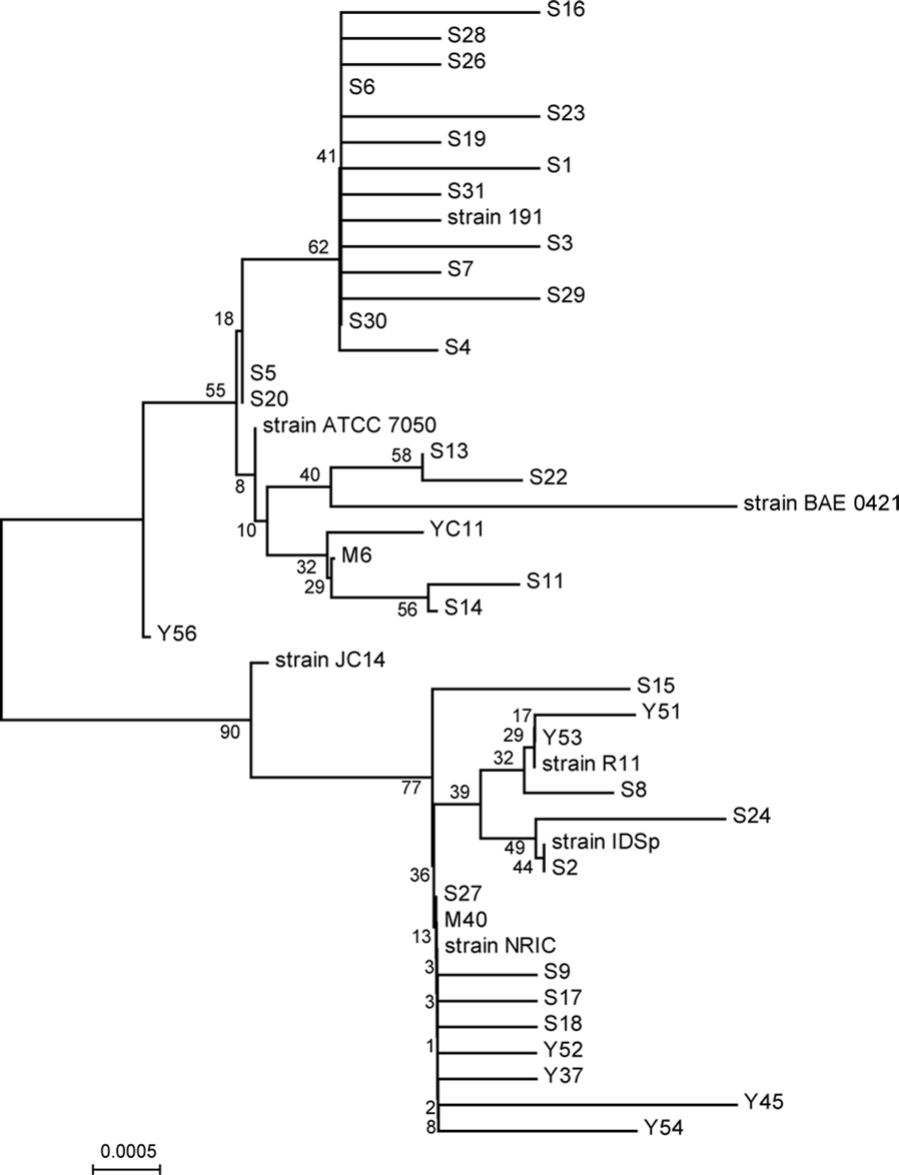
Phylogenetic tree generated using neighbor-joining analysis based on 16S rRNA gene sequences of 36 strains and other reported *B. coagulans* strains. Numbers on the branches indicate bootstrap values

In order to obtain *Bacillus coagulans* isolates with excellent performance, we firstly isolated *B. coagulans* strains from fecal samples of cows and sheep. 36 strains of *Bacillus coagulans* were obtained by isolation screening and identification. Whereafter, the 16S rRNA gene sequences of seven representative *B. coagulans* strains were extracted from the NCBI database for constructing a phylogenetic tree along with that of the 36 isolated *B. coagulans* strains (Fig. 1). The newly isolated strains form a relatively close linkage with *B. coagulans* strain 191, ATCC 7050 and R11, which reported with excellent fermentation performance and probiotic properties.

### Identification of FG degrading strains

Subsequently, we compared the physiological characteristics of all 36 *B. coagulans* strains including amylase activity, cellulase production, sugar utilization ability, spore formation ability and FG detoxification efficiency. All 36 strains, most strains can produce amylase shown by the circles stained with iodine solution on YPD-starch plates (Additional file 1: Table S1, Fig. S1). However, to cellulase activiy, only S6, S17, S24, and Y53 showed weak cellulase secretion ability (Additional file 1: Table S1, Fig. S1). Among them, strains S6, S17, Y53, Y56 were able to utilize all 11 tested sugars to produce acids (Additional file 1: Table S1).

**Fig. 2:**
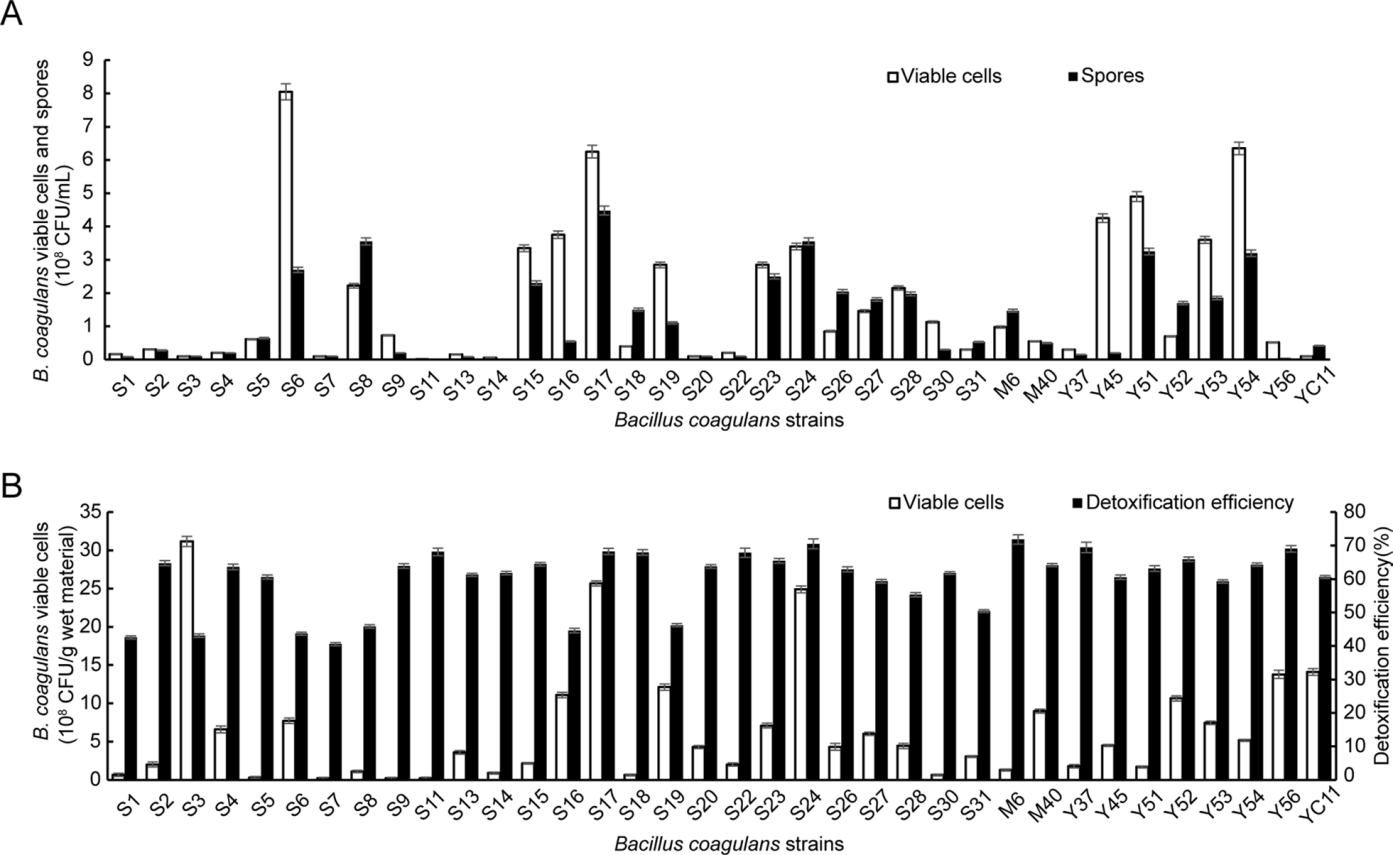
Characterization of FG degrading *B. cogulans* strains. **(A)** The concentration of viable cells and spores of *B. coagulans* after 60 h of shaking in the basal sporulation medium. **(B)** The concentration of viable cell and FG detoxification efficiency in solid-state fermentation of CSM using the *B. coagulans* strains. Data are expressed as the mean ± SD with three technical replicates

We further studied the growth and spore formation abilities of all isolated strains at flask level in the basal sporulation medium. We found that strains S6, S17, Y51, and Y54 showed higher viable cell and spore concentrations, compared with other isolated strains (Fig. 2A). Moreover, in solid-state fermentation of CSM at the jar can level, we found that all strains could degrade FG in CSM at the degradation efficiencies from 40.58 to 71.83% (Fig. 2B). Importantly, strain S17 showed higher FG detoxification efficiency and viable cell concentration in the solid-state fermentation (Fig. 2B). Consider the fermentation capacity of the strain, strain S17 was selected for further fermentation optimization to degrade the FG and enrich crude protein content in CSM. And the strain S17 has been deposited in the China Center for Type Culture Collection, the preservation number is CCTCC M 2,021,965.

### Optimal conditions for solid-state fermentation of CSM by *B. coagulans* S17

**Fig. 3:**
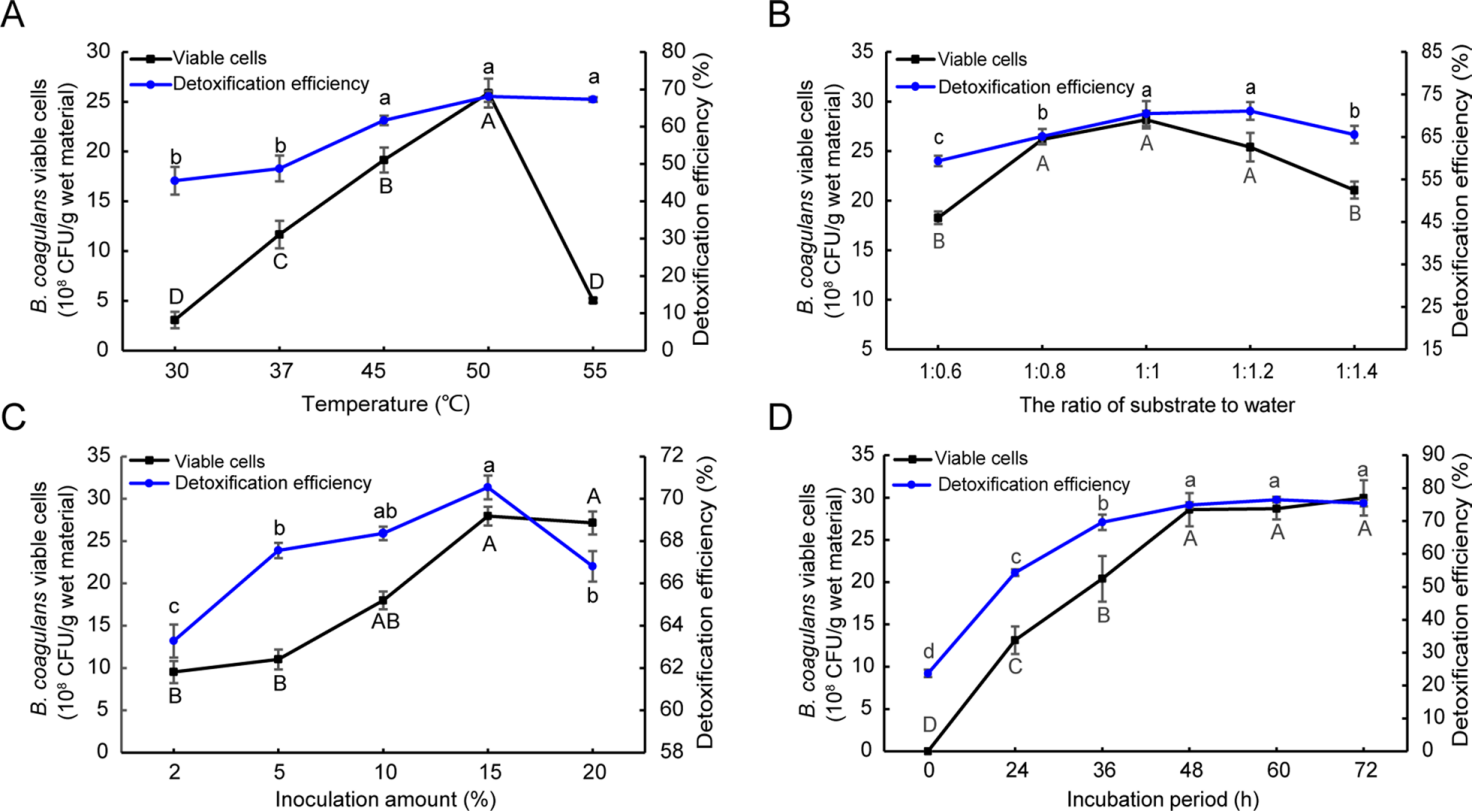
Characterization of viable cells and FG detoxification efficiencies in solid-state fermentation of CSM by *B. coagulans* S17 at different temperatures (**A**), substrate-to-water ratio (**B**), inoculation amounts (**C**) and incubation time (**D**). Data are expressed as the mean ± SD with three technical replicates. Capital and lowercase indicate the significant difference (*p* < 0.05)

To explore the best fermentation conditions, four factors (temperature, substrate-to-water ratio, inoculation amount and fermentation time) were investigated in solid-state fermentation of CSM by *B. coagulans* S17. Five fermentation temperatures (30, 37, 45, 50 and 55 °C) were used to determine their impact on FG degradation and cell growth. The results showed that the viable bacteria concentration and FG detoxification efficiency both increased with elevated temperature, reaching a maximum at 50 °C with 2.60 × 10^9^ CFU/g wet material and 68.08%, respectively (Fig. 3A).

However, higher temperature (55 °C) slightly increased FG detoxification efficiency, but sharply decreased the viable cell concentration (Fig. 3A). Therefore, 50 °C was selected as the subsequent fermentation temperature. In addition, the substrate-to-water was set as 1:0.6, 1:0.8, 1:1, 1:1.2 and 1:1.4 for the solid-state fermentation, and we found that a maximum viable cell concentration of 2.80 × 10^9^ CFU/g wet material and a highest FG detoxification efficiency of 70.50% were reached at the substrate-to-water 1:1 (Fig. 3B).

Taking into account the inoculation amount is also an important factor affecting fermentation, we used different inoculation amounts (2%-20%, v/w) to study their effects on FG detoxification and cell growth. High inoculation volume elevated FG detoxification efficiency and cell growth (Fig. 3C), and 15% (v/w) inoculation volume reached the maximum viable bacteria concentration (2.80 × 10^9^ CFU/g wet material) and FG detoxification efficiency (70.54%) (Fig. 3C). At last, the effects of fermentation time on the solid-state fermentation were also determined. Extension of fermentation time (0–48 h) significantly elevated FG detoxification efficiency and cell growth (Fig. 3D). At 48 h, the viable cell concentration and detoxification efficiency reached 2.90 × 10^9^ CFU/g wet material and 74.90%, respectively (Fig. 3D). However, further extension of fermentation time (48–72 h) showed no effect (Fig. 3D). It should be noticed that the 23.70% detoxification efficiency at 0 h might be probably caused by autoclaving.

### Optimal medium components for solid-state fermentation of CSM

**Fig. 4:**
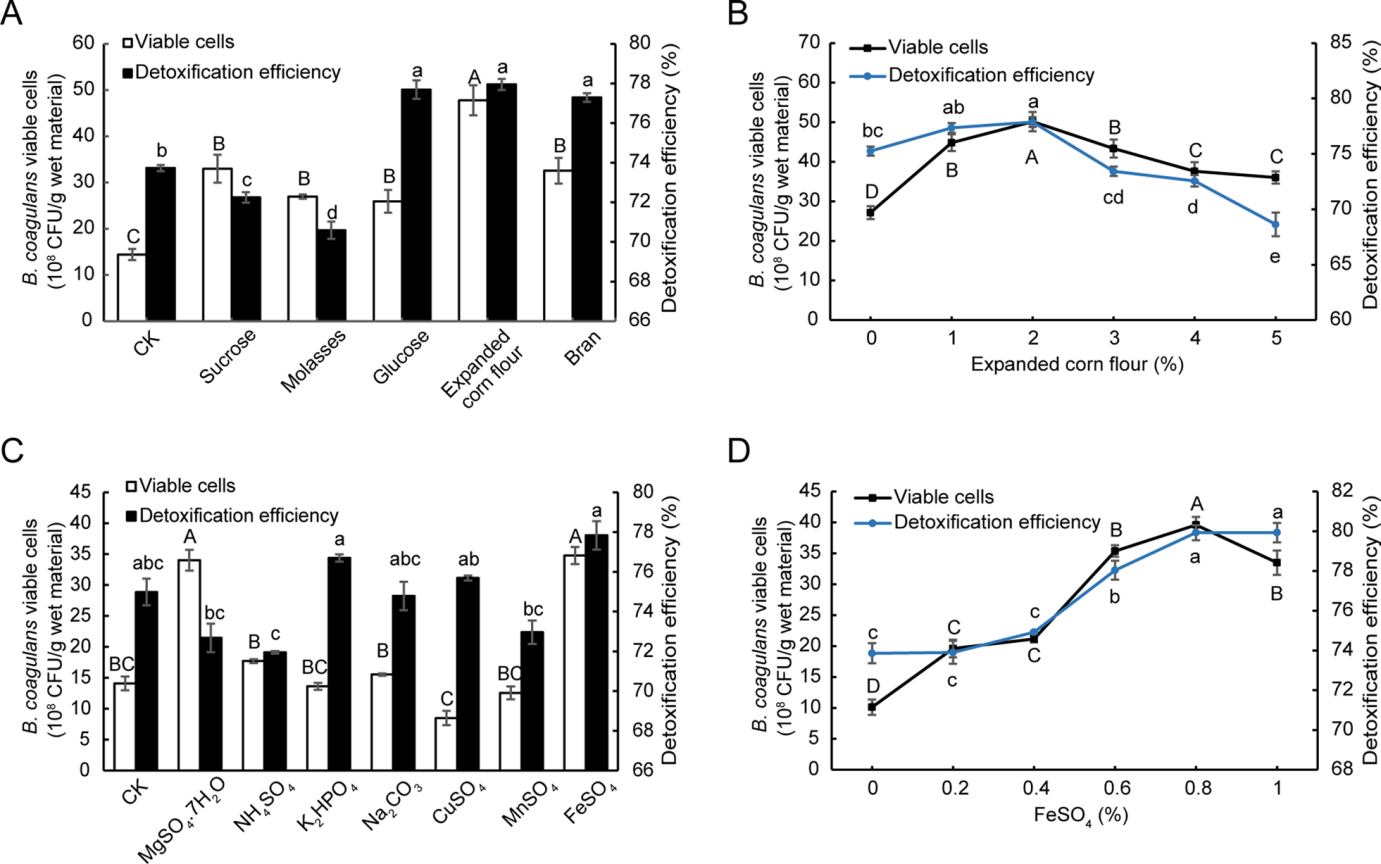
The effect of different carbon sources and inorganic salts on cell growth and FG detoxification in solid state fermentation of CSM by *B. coagulans* S17. **(A)** Different carbon sources; **(B)** Different concentrations of expanded corn flour; **(C)** Different inorganic salts; (**D)** Different concentrations of FeSO_4_. Data are expressed as the mean ± SD with three technical replicates. Capital and lowercase indicate the significant difference (*p* < 0.05)

Furthermore, we studied the effects of addition of different carbon sources (glucose, sucrose, molasses, bran, or expanded corn flour, 1% final concentration) on FG degradation and viable cell concentration. Because small molecule proteins, small peptides and free amino acids were presented in CSM [27], additional nitrogen source was not considered. Compared with no additional carbon source, adding five carbon sources significantly elevated the concentration of *B. coagulans* S17 viable cells (Fig. 4A). Interestingly, addition of expanded corn flour showed best effect. Moreover, addition of glucose, bran, and expanded corn flour significantly enhanced the FG detoxification efficiency (Fig. 4A). Consider all of the above, expanded corn flour was selected as the optimal carbon source. We further found that addition of 2% expanded corn flour showed best detoxification efficiency and cell growth in CSM fermentation (Fig. 4B).

The effect of addition of different inorganic salts (MgSO_4_·7H_2_O, NH_4_SO_4_, K_2_HPO_4_, NaCO_3_, CuSO_4_, MnSO_4_ or FeSO_4_) on CSM fermentation was also studied. We found that addition of MgSO_4_·7H_2_O and FeSO_4_ significantly increased the viable cell concentration (Fig. 4C). While, addition of NH_4_SO_4_, K_2_HPO_4_, NaCO_3_, CuSO_4_ and MnSO_4_ had no significant effect on the growth of *B. coagulans* S17 cells (Fig. 4B). Moreover, addition of K_2_HPO_4_ and FeSO_4_ significantly elevated FG detoxification efficiency in CSM fermentation (Fig. 4C). Hence, FeSO_4_ was selected as the inorganic additive (Fig. 4C). We further found that addition of 0.8% FeSO_4_ showed the best effect on FG detoxification the *B. coagulans* growth (Fig. 4D).

### Box-Behnken test for CSM fermentation and significance test analysis

To obtain better fermented conditions, the Box-Behnken response surface method was used to optimize the solid-state fermentation of CSM. Firstly, due to the temperature will increase during the fermentation process with the increase of the amount of stacking material after industrial scale-up, the initial fermentation temperature during industrial scale-up should not be too high. Therefore, the factors in the response surface do not consider temperature. The Box-Behnken test was designed using Design Expert software to quantify the effectiveness of key factors (substrate-to-water ratio, A; inoculation amount, B; fermentation time, C; the content of expanded corn flour, D; and FeSO_4_, E) and the interactions among them. For this test, the viable *B. coagulans* cell concentration and FG detoxification efficiency were selected as the response value Y_1_ and Y_2_. The independent variables, codes and levels are shown in Additional file 1: Table S2. 43 experiments were carried out to test the concentration of viable *B. coagulans* cells and FG detoxification efficiency under the Box-Behnken experimental design (Table 1). The minimum and maximum the concentration of viable cells were 1.10 and 7.45 × 10^9^ CFU/g wet material, respectively. While, the minimum and maximum detoxification efficiencies were 75.77% and 87.71%, respectively (Table 1).

**Table 1:** The experimental results of Box-Behnken design and corresponding schemes

Treatment	Factor					Y_1_ (10^9^ CFU/g wet material)	Y_2_ (%)
	A	B	C	D	E		
1	0.75	10	48	2	0.8	2.47	82.30
2	1.25	10	48	2	0.8	2.80	81.38
3	0.75	20	48	2	0.8	5.93	81.64
4	1.25	20	48	2	0.8	4.65	82.96
5	1.00	15	36	1	0.8	1.57	82.83
6	1.00	15	60	1	0.8	6.02	84.61
7	1.00	15	36	3	0.8	1.98	82.43
8	1.00	15	60	3	0.8	4.40	81.84
9	1.00	10	48	2	0.6	5.98	78.48
10	1.00	20	48	2	0.6	5.01	82.70
11	1.00	10	48	2	1.0	2.09	87.64
12	1.00	20	48	2	1.0	2.69	87.71
13	0.75	15	36	2	0.8	2.51	81.51
14	1.25	15	36	2	0.8	1.46	83.68
15	0.75	15	60	2	0.8	5.57	81.84
16	1.25	15	60	2	0.8	4.36	82.23
17	1.00	15	48	1	0.6	1.90	79.20
18	1.00	15	48	3	0.6	4.12	76.90
19	1.00	15	48	1	1.0	3.93	86.52
20	1.00	15	48	3	1.0	2.94	87.38
21	1.00	10	36	2	0.8	1.39	83.82
22	1.00	20	36	2	0.8	2.47	79.99
23	1.00	10	60	2	0.8	4.63	82.43
24	1.00	20	60	2	0.8	4.87	82.56
25	0.75	15	48	1	0.8	2.03	78.35
26	1.25	15	48	1	0.8	4.10	82.37
27	0.75	15	48	3	0.8	5.93	83.29
28	1.25	15	48	3	0.8	3.11	82.83
29	1.00	15	36	2	0.6	1.99	75.77
30	1.00	15	60	2	0.6	5.26	77.16
31	1.00	15	36	2	1.0	1.10	84.81
32	1.00	15	60	2	1.0	6.64	86.19
33	0.75	15	48	2	0.6	3.47	79.07
34	1.25	15	48	2	0.6	4.21	78.74
35	0.75	15	48	2	1.0	4.54	87.18
36	1.25	15	48	2	1.0	5.64	84.41
37	1.00	10	48	1	0.8	3.85	85.40
38	1.00	20	48	1	0.8	3.17	85.27
39	1.00	10	48	3	0.8	5.95	85.86
40	1.00	20	48	3	0.8	2.81	85.40
41	1.00	15	48	2	0.8	7.45	85.66
42	1.00	15	48	2	0.8	6.97	85.33
43	1.00	15	48	2	0.8	6.83	86.19

The concentrations of viable cells and detoxification efficiencies were selected as the response value Y_1_ and Y_2_. The independent variables A, B, C, D and E were corresponding to material to water, inoculation amount, incubation period, the content of expanded corn flour and FeSO_4_ are shown in the table

Considering the feasibility of the Box-Behnken experimental model, we first performed an ANOVA to Box-Behnken experimental design (Additional file 1: Tables S3 and S4). Adequate Precision was used to measure the signal-to-noise ratio with a value > 4 being desirable.

In this study, when the viable *B. coagulans* cell concentration and FG detoxification efficiency were the corresponding values, the coefficient of variation (CV) were 29.65% and 1.95%, the overall standard deviation (SD) were 1.18 and 1.61, and the Adequate Precision were 7.68 and 10.27, respectively (Additional file 1: Tables S3 and S4). The results were analyzed using multiple regression fitting, and the quadratic polynomial regression equation of Y_1_ (the viable *B. coagulans* cell concentration) and Y_2_ (FG detoxification efficiency) on coding independent variables A, B, C, D and E was obtained. The results showed that the experimental data for Y_1_ (*p* = 0.0028, R^2^ = 0.7596) and Y_2_ (*p* < 0.0001, R^2^ = 0.8542) were statistically significant (Additional file 1: Tables S3 and S4). The significant factors were incubation period (C, *p* < 0.0001) to Y_1_ and FeSO_4_ content (E, *p* < 0.0001) to Y_2._

$$\begin{aligned} \left( {{\text{Y}}_{{1}} \times {1}0^{{9}} } \right) \, = & \, - {116}.{93} + {58}.{\text{89A}} + {2}.{\text{37B}} + {1}.{\text{42C}} + {19}.{\text{63D}} + {49}.{5}0{\text{E}} - 0.{\text{32AB}} \\ & \quad - 0.0{\text{1AC}} - {4}.{\text{89AD}} + {1}.{\text{8AE}} - 0.00{\text{4BC}} - 0.{\text{12BD}} + 0.{\text{39BE}} - 0.0{\text{4CD}} \\ & \quad + 0.{\text{24CE}} - {4}.0{\text{1DE}} - {22}.{\text{81A}}^{{2}} - 0.0{\text{6B}}^{{2}} - 0.0{\text{1C}}^{{2}} - {1}.{\text{84D}}^{{2}} - {38}.{\text{28E}}^{{2}} \\ \end{aligned}$$

$$\begin{aligned} \left( {{\text{Y}}_{{2}} \% } \right) \, = & \, { 85}.{73} + 0.{\text{21A}} + 0.0{\text{58B}} + 0.{\text{25C}} + 0.0{\text{87D}} + {3}.{\text{99E}} + 0.{\text{56AB}} - 0.{\text{44AC}} \\ & \quad - {1}.{\text{12AD}} - 0.{\text{61AE}} + 0.{\text{99BC}} - 0.0{\text{82BD}} - {1}.0{\text{4BE}} - 0.{\text{59CD}} + {\text{CE}} + 0.{\text{79DE}} \\ & \quad - {2}.{27}^{{2}} - 0.{\text{46B}}^{{2}} - {2}.{\text{28C}}^{{2}} - 0.{\text{88D}}^{{2}} - {1}.{\text{77E}}^{{2}} \\ \end{aligned}$$

### Response surface analysis of CSM fermentation

In order to obtain the response range for the five factors studied, three of the variables were fixed at the central value, and the effects of the other two variables on the viable *B. coagulans* cell concentration and FG detoxification efficiency were analyzed and evaluated based on the response surface diagram and contour plot of the multivariate quadratic equation (Additional file 1: Fig. S2A).

When the viable cell concentration was the corresponding value, results of the response surface graph and contour plot showed that factor C (fermentation time) had a significant effect on viable cell concentration (*p* < 0.0001), and the interaction between factor A and D was also significant (*p* = 0.0498) (Additional file 1: Fig. S2, Table S3). The interaction between the other factors was not significant difference (*p* > 0.05) (Additional file 1: Table S3). When the FG detoxification efficiency was the corresponding value, results of the response surface graph and contour plot showed that factor E (FeSO_4_ content) had a meaningful effect on FG detoxification efficiency (*p* < 0.0001), and the interaction between the other factors was not significant difference (Additional file 1: Table S4).

The predicted maximum of viable cell concentration and FG detoxification efficiency were 7.16 × 10^9^ CFU/g wet material and 86.97%, respectively, under the following conditions: the substrate-to-water ration was 0.99, inoculation amount was 15.32%, fermentation time was 52 h, the expanded corn flour content was 1.98% and the FeSO_4_ content was 0.89%. The predicted value was higher than the actual maximum value in the Box-Behnken experiment.

To verify the accuracy of the model, three experiments with repeating five times were conducted using the predicted optimal conditions. Box-Behnken model validation experiments found that the maximum viable *B. coagulans* cell concentration was 6.42 ± 0.31 × 10^9^ CFU/g wet material. At this time, the spore concentration was 1.16 ± 0.84 × 10^10^ CFU/g dry material, and the maximum detoxification efficiency of CSM was 86.01 ± 1.09% (Table 2). After fermentation under the optimal conditions, the FG content in CSM decreased from 923.80 ± 11.30 mg/kg to 129.20 ± 10.03 mg/kg, and the crude protein content increased from 47.98 ± 0.89 to 52.13 ± 0.93%.

**Table 2:** Summary of verification experiments

	Viable cells (10^9^ CFU/g wet material)	Spores (10^10^ CFU/g dry material)	FG (mg/kg)	Detoxification efficiency (%)	Crude Protein (%)
Initial CSM	0	0	923.80 ± 11.30	0	47.98 ± 0.89
Box-Behnken model validation	6.42 ± 0.31	1.16 ± 0.84	129.20 ± 10.03	86.01 ± 1.09	52.13 ± 0.93
Scale-up verification	4.29 ± 0.37	0.83 ± 0.72	241.02 ± 7.69	73.91 ± 0.83	50.19 ± 0.65
Scale-up and optimized verification	6.67 ± 0.34	1.68 ± 2.81	167.90 ± 12.09	81.83 ± 1.31	52.82 ± 1.05

### Further optimization of CSM fermentation at a scale-up level

According to the above optimal conditions for solid-state fermentation under laboratory conditions, except the fermentation temperature, scale-up verification experiments are carried out in a windrow with 60 cm (length) × 35 cm (width) × 10 cm (height) in a fermentation workshop at 40 °C fermentations. The viable *B. coagulans* cell concentration was 4.29 ± 0.37 × 10^9^ CFU/g wet material, and the spore concentration was 8.28 ± 0.72 × 10^9^ CFU/g dry material. The FG detoxification efficiency was 73.91 ± 0.83%, and the crude protein content of CSM increased from 47.98 ± 0.89% to 50.19 ± 0.65% (Table 2). The increase of crude protein content was probably due to utilization of the polysaccharides and oligosaccharides in the CSM by *B. coagulans* S17 during fermentation. The lower FG detoxification efficiency and viable cell concentration obtained in the scale-up experiment were due to the fact that the fermentation temperature was lower than the optimal condition and hard to control.

**Fig. 5:**
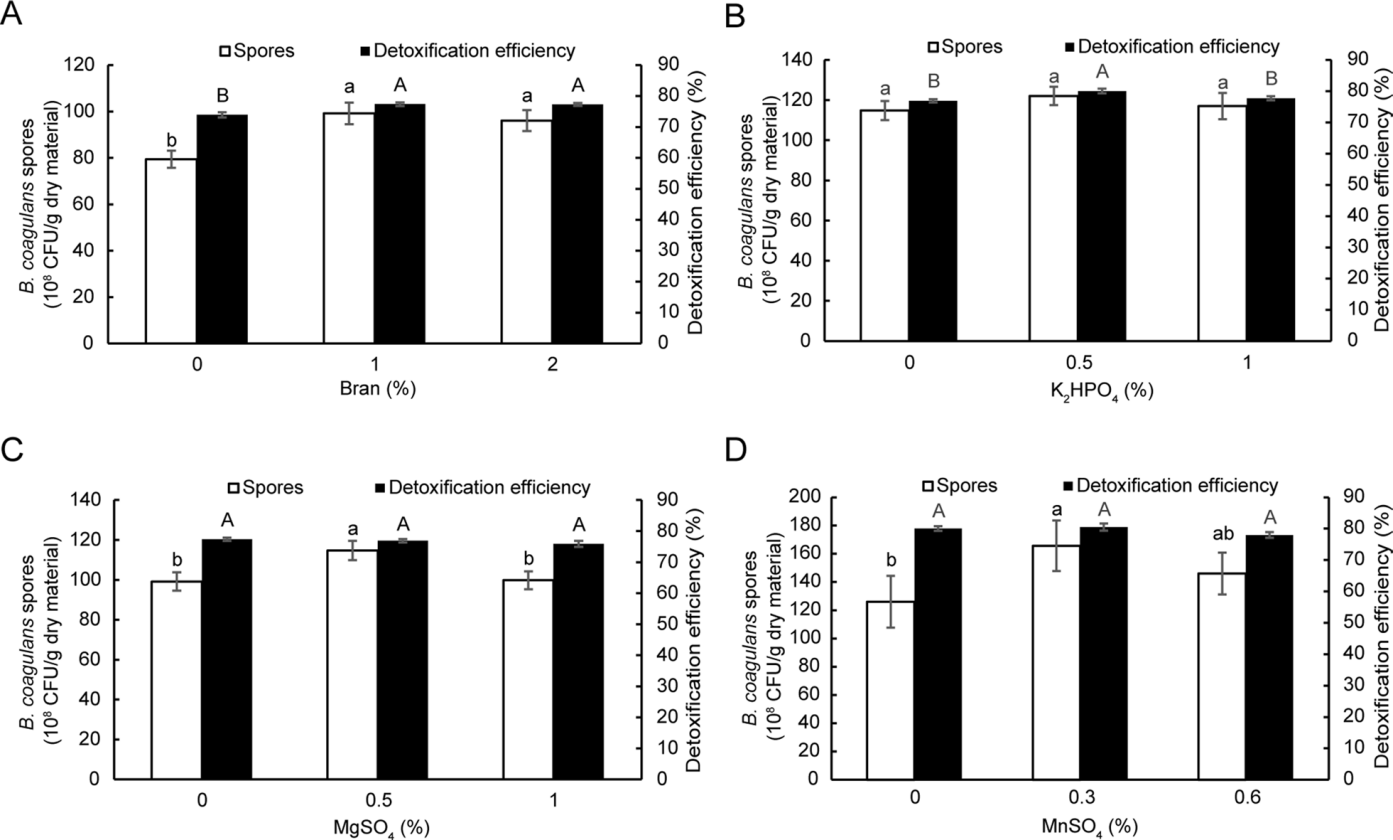
Optimization of CSM fermentation at a scale-up level. The effect of the content of bran (**A**), K_2_HPO_4_ (**B**), MgSO_4_ (**C**) and MnSO_4_ (**D**) on spore formation and FG detoxification in solid-state fermentation of CSM. Data are expressed as the mean ± SD with three technical replicates. Capital and lowercase indicate the significant difference (*p* < 0.05)

In order to improve the fermentation performance, the effect of the content of bran, MgSO_4_, K_2_HPO_4_ and MnSO_4_ on CSM fermentation was investigated. We found that addition of 1% bran and 0.5% K_2_HPO_4_ significantly enhanced both FG detoxification efficiency and spore formation (Fig. 5A and B ), while addition of 0.5% MgSO_4_ and 0.3% MnSO_4_ increased spore formation (Fig. 5C and D ). These four factors were included in the above optimized fermentation conditions, the scale-up and optimized validation experiments resulted in the viable *B. coagulans* cell concentration of 6.67 ± 0.34 × 10^9^ CFU/g wet material, the spore concentration of 1.68 ± 2.81 × 10^10^ CFU/g dry material, and FG detoxification efficiency of 81.83 ± 1.31% (Table 2). Meanwhile, the crude protein content of CSM increased from 47.98 ± 0.89% to 52.82 ± 1.05%. The results of the scale-up and optimized verification experiments were significantly better than the results of the scale-up verification experiments in all aspects (Table 2). Compared with the Box-Behnken model validation experiments, except the detoxification efficiency, the results were also better in other aspects.

## Discussion

Microbial solid-state fermentation is an important method to degrade FG in the processing of cotton by-products [28]. Previous studies have reported that *Bacillus*, yeast and lactic acid bacteria were used as the fermentation strains of CSM. However, fermentation using yeast might produce alcohol in anerobic conditions [16]. While fermentation through *Bacillus subtilis* affected fermentation flavor [15] and through *Lactobacillus* species poorly improved the nutritional value of CSM [18]. In this study, we isolated 36 strains of *B. coagulans* strains. These strains form a relatively close linkage with *B. coagulans* strain 191, ATCC 7050 or R11. *B. coagulans* strain 191 was reported to utilize most sugars and catalyzed the conversion of leaves into lactic acid at a productivity of 1.56 g/L/h [29]. Strain ATCC 7050 showed probiotic effects on growth performance improvements, antioxidant activity and increased survival rate against *Vibrio parahaemolyticus* attack in South American white shrimp [30]. Strain R11 was found to prevent the lead adsorption in gut cells through lead ion binding and gut microbiota regulation [31, 32]. Therefore, the newly isolated *B. coagulans* strains might have good fermentation and probiotic abilities.

Among these isolated strains, S17 showed best fermentation performance and FG degradation ability Therefore, we used strain S17 in the solid-state fermentation for degradation of FG. The fermentation temperature was set at 50 °C, which is the optimal growth temperature for *B. coagulans* strains [22] and could inhibit the growth of other bacteria and fungi [25]. 1:1 of substrate-to-water ratio was used in this study. Lower moisture content in solid-state fermentation reduced the solubility of nutrients of the substrate and degree of swelling [33] and increased water loss due to quick volatilization during fermentation.

In this study, the fermentation process was optimized by single factor and Box-Behnken experiments, and further verified by scale-up experiments. After fermentation, the FG content in CSM is reduced to 167.90 mg/kg with a detoxification efficiency of 81.82%, the crude proteins increased by 4.83%, and the spore concentration of strain S17 reaches 1.68 × 10^10^ CFU/g dry material. Our process using *B. coagulans* strain S17 operates with a short fermentation time and the anaerobic condition, which showed obvious advantages over the reported processes using *B. subtilis*, *Lactobacillus* species and fungi. According to the previous reports, 79.00% of FG was degraded using *B. subtilis* for 72 h, and the crude protein was increased by 4.80% [34]. *Lactobacillus agilis* WWK129 reduced FG in CSM by about 80% and increased crude protein content by about 4% after 5 days of anaerobic fermentation [35]. Moreover, FG was degraded by 96.67%, and the crude protein content increased by about 12.36% in the CSM fermentation for 48 h using *Candida tropicalis* ZD-3 [36]. However, 74.00% detoxification efficiency at 0 h was found in that study, that might be probably caused by autoclaving [36].

Numerous studies have reported beneficial effects of *B. coagulans* on human and animal health [37]. And it produces large amounts of lactic acid and other metabolites that can reduce the risk of pathogenic infection in the gut [26]. The lactic acid in the fermented CSM feed can increase the acidity of the feed, inhibit the growth of bacteria that make the feed spoil, and delay and prevent the feed from mildew [23]. And *B. coagulans* also has spores with good heat resistance, stability and stress resistance [19]. Therefore, using *B. coagulans* for solid-state fermentation of CSM, it can not only reduce the toxicity of FG, improve the nutritional value of CSM, but also prevent *E. coli* and *Staphylococcus* infections in gastrointestinal system and significantly improves the development of animals (such as poultry, pigs, etc.) by increasing digestibility [38]. In summary, our study establishes a feasible process for FG degradation, *B. coagulans* spore production and crude protein enrichment in solid-state fermentation of CSM through *B. coagulans* S17, and the fermented CSM could be used as a good feed material for breeding industry.

## Conclusions

In this study, the optimal components in CSM fermentation consists of 2% of expanded corn flour, 1% of bran, 0.8% of FeSO_4_, 0.5% of MgSO_4_, 0.5% of K_2_HPO_4_ and 0.3% of MnSO_4_ under the optimal fermentation conditions: fermentation at 40 °C with inoculum amount of 15% (v/w), material-to-water ratio of 1:1 and pH in nature for 52 h. After fermentation, the FG content in CSM is reduced with a detoxification efficiency of 81.83%, the crude proteins increased by 4.83%, and the spore concentration of *B. coagulans* S17 reaches 1.68 × 10^10^ CFU/g. This study establishes a feasible process for FG degradation, *B. coagulans* spore production and crude protein enrichment in solid-state fermentation of CSM through *B. coagulans* S17.

## Materials and methods

### Materials and medium

The CSM used in this study is from Xin jiang Aksu New Grain Oil Co., Ltd. YPD medium (20 g/L glucose, 10 g/L yeast and 20 g/L tryptone) was used for seed culture. Fecal samples used for *B. coagulans* isolation were collected in Mianzhu county. Soluble starch medium or cellulose medium agar plate (10 g/L tryptone, 5 g/L NaCl, 4 g/L K_2_HPO_4_, 15 g/L agar and 2 g/L soluble starch or 10 g/L CMC-Na) was used to test the growth ability of the isolated strains. Different sugars (glucose, xylose, sucrose, lactose, arabinose, maltose, sorbitol, galactose, raffinose and stachyose) were used to replace the glucose in YPD medium to test the sugar utilization ability.

### Measurement of crude proteins and free gossypol

The dry matter (DM) content was measured by drying at 65 °C for 5 h. The crude protein content was determined by Kjeldahl method [39]. FG was determined by the official method of the American Oil Chemists Society [40].

### Isolation and characterization of *B. coagulans*

The fecal samples were diluted with 90 mL of sterile water and shaken for 30 min at room temperature, and then were filtered through sterile. The filtrate was heat-treated in a water bath at 80 °C for 15 min. After gradient dilution, the filtrate was plated on the YPD plates containing bromocresol violet at 50 °C for 24 h. The colonies with yellow discolored circles were selected for further characterization. The genomic DNA of the isolated strains were extracted using the TIANamp Bacteria DNA Kit (Tiangan, Beijing, China). The 16S rDNA sequence was amplified by PCR using the universal primers 27f and 1492r, and PCR products were analyzed by agarose-electrophoresis, then purified using the E.Z.N.A. Cycle-Pure Kit (Norcross, Georgia, USA), and sequenced by Invitrogen (Chengdu, China). The 16S rDNA sequences were analyzed against the NCBI database through BLAST. The 16S rRNA gene sequences of seven representative *B. coagulans* strains (191, ATCC7050, BAE0421, JC14, R11, IDSp and NRIC) with accession numbers KY486267.1, NR115727.1, FJ392315.1, LC337958.1, MF539755.1, AF466695.1 and AB362709.1 were extracted from the NCBI database, and a phylogenetic tree was constructed using the neighbor-joining method with MEGA6 [41].

### Identification of physiological characteristics of *B. coagulans*

Bacterial culture (2.5 μL for each sample) was spotted on the soluble starch medium plate, cellulose medium plate, or sugar plate containing bromocresol violet, and incubated at 50 °C for 24 h. Starch and CMC-Na unitizations were determined by iodine staining for 30 s and Congo red staining for 10 min–15 min, respectively. To compare the lactic acid production efficiencies, the parameter of diameter of straining circle/colony circle was used. Spore formation was conducted by inoculating 1% *B. coagulans* cultures into the basic sporulation medium (5 g/L tryptone, 2 g/L beef extract, 3 g/L yeast, 5 g/L NaCl, 0.005 g/L MgSO_4_·7H_2_O, 0.02 g/L MnSO_4_·H_2_O) and incubated in a shaker at 50 °C at 250 r/min for 60 h. The viable cells were calculated by plating on YPD medium, and spore concentration was calculated by plating of cell culture after heat treatment at 80 °C for 15 min.

### Optimization of solid-state fermentation conditions

Single factors including incubation temperature, the ratio of substrate-to-water, inoculation amount, incubation period, carbon sources and minerals additives were initially optimized in can jars with 500 mL under aerobic fermentation. The original pH of the cottonseed meal was used as the initial pH of the fermentation and was not adjusted. CSM is crushed through a 60-mesh sieve and sterilized at 121 °C for 30 min. The initial condition included 15% inoculum amount, 1:0.8 of the substrate-to-water ratio at 50 °C and the original pH for 40 h. The optimal culture temperature (30 °C, 37 °C, 45 °C, 50 °C and 55 °C), substrate-to-water ratio (1:0.6, 1:0.8, 1:1, 1:1.2 and 1:1.4), inoculation amount (2%, 5%, 10%, 15%, and 20%) and fermentation time (24 h, 36 h, 48 h, 60 h and 72 h) were analyzed, according to the detoxification efficiency of the CSM and viable cell concentration of *B. coagulans* S17 after fermentation. The optimal carbon source was identified through addition of 1% sucrose, molasses, glucose, expanded corn flour, or bran to compare detoxification efficiency and viable cell concentration. Added 0%, 1%, 2%, 3%, 4%, and 5% of the best carbon source selected by the above method to CSM, and determined the best carbon source addition amount according to the fermentation effects. The optimal inorganic salt was identified through addition of 0.5% MgSO_4_·7H_2_O, NH_4_SO_4_, K_2_HPO_4_, NaCO_3_, CuSO_4_, MnSO_4_ or FeSO_4_ to compare detoxification efficiency and viable cell concentration. Added 0%, 0.2%, 0.4%, 0.6%, 0.8%, 1% of the best inorganic salt selected by the above method to CSM, and determined the best amount of inorganic salt added according to the fermentation effect. All experiments were done in triplicate.

### Box-Behnken experimental design

Based on the single-factor experiment, the optimal conditions for solid-state fermentation of CSM were obtained using the Box–Behnken response surface (Design-Expert 12) method. The Box-Behnken experimental design is based on the mathematical model:

$${\text{Y}} = {\upbeta }_{0} + \sum {\upbeta }_{{\text{i}}} {\text{x}}_{{\text{i}}} + \sum {\text{x}}_{{\text{i}}} {\text{x}}_{{\text{j}}} + \sum {\upbeta }_{{{\text{ii}}}} {\text{x}}_{{\text{i}}}^{{2}}$$

Y is the response value (the FG detoxification efficiency and viable bacteria concentration); β_0_ is a constant term, β_i_ and β_ii_ are regression coefficients, and x_i_ and x_j_ are coded variables (the substrate-to-water ratio, inoculation amount, fermentation time, the content of expanded corn flour and FeSO_4_ [42]. Each factor in the Box-Behnken design was coded as three levels with − 1, 0, and + 1 (Additional file 1: Table S1). The specific experimental conditions, automatically given by the software to set the conditions, are shown in Table 2. The quadratic regression fitting was conducted to get the quadratic equation with interactive terms and square terms using the corresponding code. The primary effects and interaction effects of each factor were analyzed. Finally, the optimal value was obtained within a certain level. One-way ANOVA analysis of multiple variables were carried out with the Design-Expert 12 [43].

### Scale-up solid-state fermentation of CSM

In the scale-up process of a 60 cm × 35 cm × 10 cm (length × width × height) window system, the fermentation temperature was adjusted to 40 °C in consideration of industrial production cost, and other fermentation conditions were the optimal fermentation process obtained in the Box–Behnken response surface experiment. Moreover, the effect of the contents of bran, MgSO_4_, K_2_HPO_4_ and MnSO_4_ on fermentation of CSM were explored.

## Supplementary Information

**Additional file 1: Figure S1.** Identification of physiological characteristics of *B. coagulans* strains. **Figure S2. **The effects of substrate-to-water ratio and the content of expanded corn flour on the concentration of viable bacterial cells.** Table S1. **Estimation of the activities of secretory enzymes and the acid-producing abilities of *B. coagulans* species using different sugars.** Table S2.** Box-Behnken experimental design. **Table S3. **ANOVA results of the quadratic model for the concentration of viable cells. **Table S4. **ANOVA results of the quadratic model for the FG detoxification rate in solid-state fermentation of CSM**.**

## Data Availability

The datasets generated during and/or analysed during the current study are available from the corresponding author on reasonable request. All data generated or analysed during this study are included in this published article (and its additional information files).
