# Supplementary material for: Effective gossypol removal from cottonseed meal through optimized solid-state fermentation by Bacillus coagulans
Source: Microb Cell Fact. 2022 Dec 1;21:252. doi: 10.1186/s12934-022-01976-1 (PMC9714218; doi:10.1186/s12934-022-01976-1)
Supplement: Supplementary file 1 — Additional file 1: Figure S1. Identification of physiological characteristics of B. coagulans strains. Figure S2. The effects of substrate-to-water ratio and the content of expanded corn flour on the concentration of viable bacterial cells. Table S1. Estimation of the activities of secretory enzymes and the acid-producing abilities of B. coagulans species using different sugars. Table S2. Box-Behnken experimental design. Table S3. ANOVA results of the quadratic model for the concentration of viable cells. Table S4. ANOVA results of the quadratic model for the FG detoxification rate in solid-state fermentation of CSM. [file 12934_2022_1976_MOESM1_ESM.docx]

**Additional Material**

**Effective gossypol removal from cottonseed meal through optimized solid-state fermentation by *Bacillus coagulans***

Zhenting Zhang^1, 2#^, Danlu Yang^1#^, Ling Liu^1^, Zhangbing Chang^1^, Nan Peng^1, 3*^

^1^ State Key Laboratory of Agricultural Microbiology, Hubei Hongshan Laboratory, College of Life Science and Technology, Huazhong Agricultural University, Wuhan, 430070, Hubei, P.R. China

^2^ School of Public Health, Guizhou Medical University, Guiyang, 550025, Guizhou, P.R. China.

^3^ National Engineering Research Center of Microbial Pesticides, 430070, Hubei, P.R. China

^#^These authors contributed equally: Zhenting Zhang and Danlu Yang.

^*^Correspondence should be addressed to Nan Peng: Tel: +86 27 8728 1040, Fax: +86 27 8728 0670, Email: [nanp@mail.hzau.edu.cn](mailto:nanp@mail.hzau.edu.cn)

**Additional Figures and tables：**


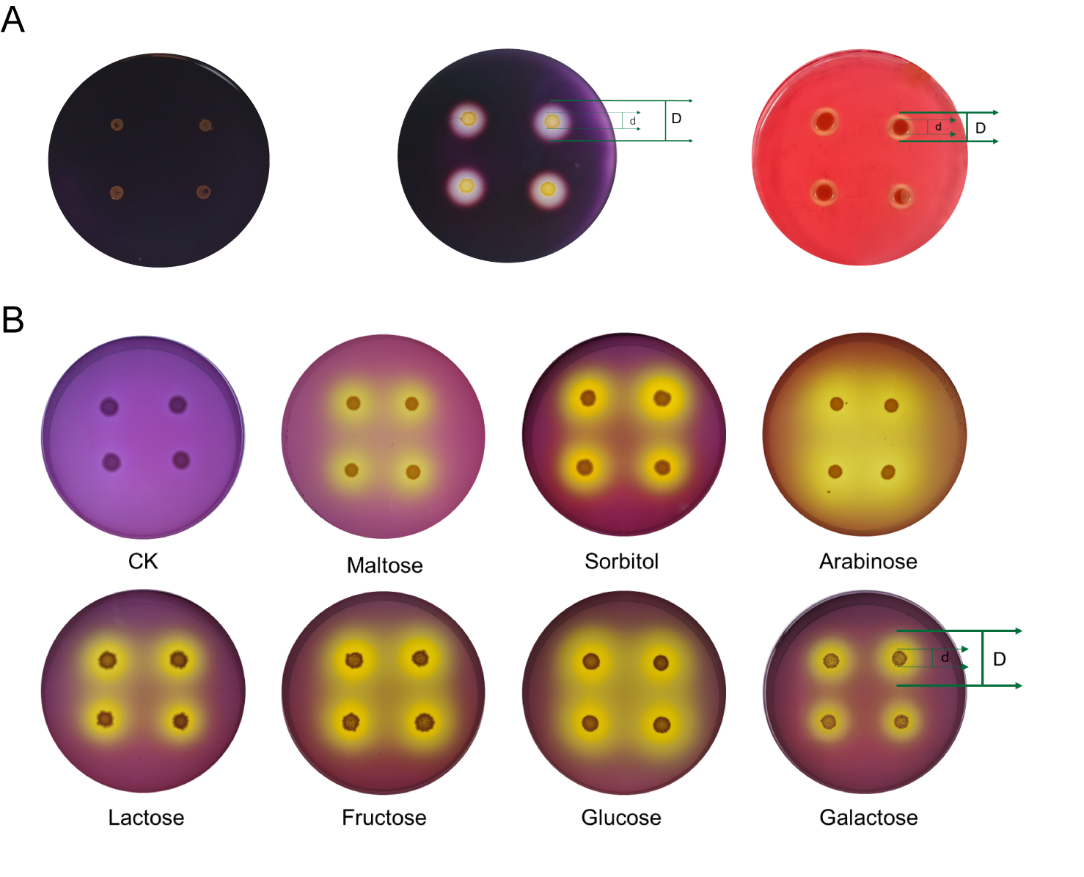


**Figure S1. Identification of physiological characteristics of *B. coagulans* strains.** (**A**) Identification of the secretase activity of *B. coagulans*. Left plate: negative control; middle plate: positive control with the colonies of the strain secretes amylase on soluble starch plate staining with iodine; Right plate: positive control that secretes cellulase on CMC-Na plate straining with Congo red. (**B**) *B. coagulans* uses different sugars to produce acids on the plates with bromocresol violet. D: diameter of transparent circle; d: colony diameter.


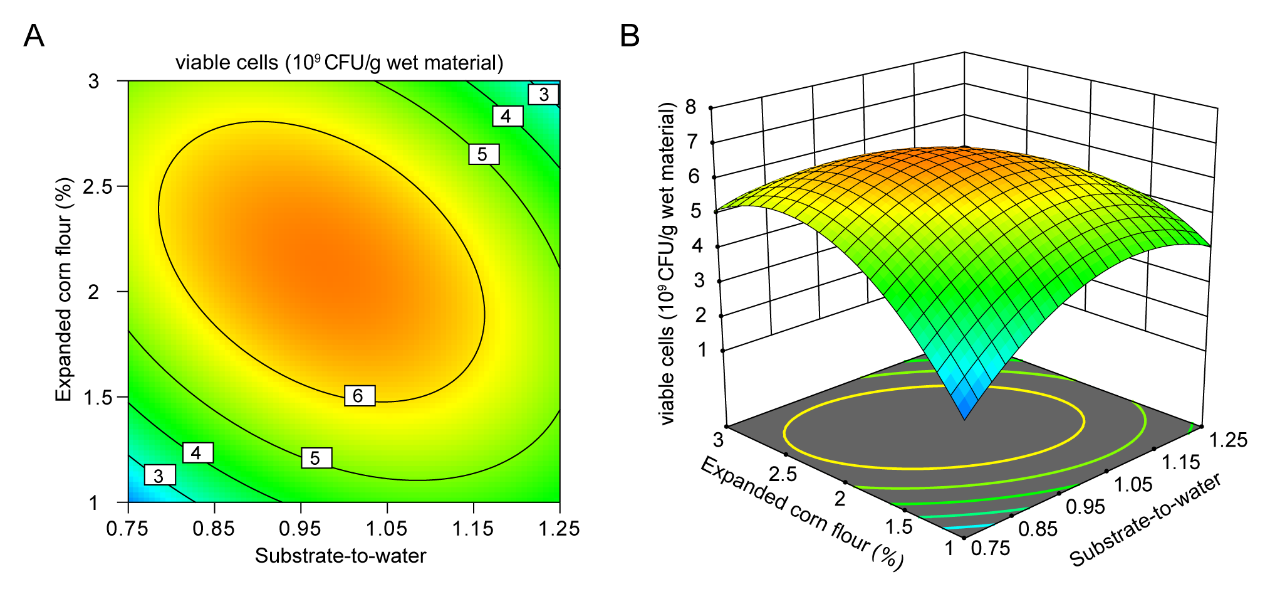


**Figure S2. The effects of** **substrate-to-water ratio and the content of expanded corn flour on the concentration of viable bacterial cells.** Contour plots (**A**) and response surface diagram (**B**) of substrate-to-water and the content of expanded corn flour was analyzed on the concentration of viable bacteria. The shape of contours could reflect the intensity of the interaction effect. The ellipse indicates that the interaction between material to water and the content of expanded corn flour on the concentration of viable bacteria is significant.

| Strains | Amylase | Cellulase | Fructose | Glucose | Xylose | Sucrose | Lactose | Arabinose | Maltose | Sorbitol | Galactose | Raffinose | Stachyose |
| --- | --- | --- | --- | --- | --- | --- | --- | --- | --- | --- | --- | --- | --- |
| S1 | ++ | - | +++ | +++ | + | +++ | - | - | ++ | - | +++ | ++ | +++ |
| S2 | ++ | - | +++ | +++ | - | - | + | - | - | ++ | +++ | - | - |
| S3 | +++ | - | +++ | +++ | ++ | - | ++ | ++ | ++ | +++ | +++ | ++ | +++ |
| S4 | +++ | - | +++ | +++ | + | +++ | - | - | ++ | - | - | ++ | +++ |
| S5 | ++ | - | +++ | +++ | - | + | ++ | - | ++ | - | +++ | - | - |
| S6 | +++ | + | +++ | +++ | ++ | +++ | ++ | + | +++ | + | +++ | +++ | +++ |
| S7 | +++ | - | +++ | +++ | + | +++ | - | - | ++ | - | +++ | ++ | +++ |
| S8 | +++ | - | +++ | +++ | ++ | +++ | - | - | +++ | - | ++ | ++ | + |
| S9 | ++ | - | +++ | +++ | ++ | - | + | ++ | ++ | ++ | +++ | - | - |
| S11 | + | - | +++ | +++ | - | + | - | - | ++ | + | + | - | - |
| S13 | - | - | ++ | +++ | - | - | - | +++ | ++ | +++ | ++ | - | - |
| S14 | + | - | +++ | +++ | - | + | - | - | ++ | - | ++ | - | - |
| S15 | ++++ | - | +++ | +++ | +++ | + | ++ | +++ | ++ | +++ | ++ | - | ++ |
| S16 | ++++ | - | +++ | +++ | ++ | +++ | - | - | +++ | + | +++ | +++ | +++ |
| S17 | + | + | +++ | +++ | +++ | + | ++ | +++ | ++ | +++ | ++ | +++ | + |
| S18 | - | - | +++ | +++ | +++ | - | +++ | +++ | ++ | + | +++ | - | - |
| S19 | ++ | - | +++ | +++ | ++ | +++ | - | - | + | - | ++ | ++ | +++ |
| S20 | + | - | +++ | +++ | - | + | ++ | - | +++ | - | +++ | - | - |
| S22 | ++++ | - | +++ | +++ | - | - | ++ | - | +++ | - | +++ | - | - |
| S23 | ++++ | - | +++ | +++ | +++ | +++ | - | - | ++ | - | +++ | ++ | ++ |
| S24 | ++ | + | +++ | +++ | +++ | + | ++ | +++ | ++ | +++ | +++ | - | - |
| S26 | +++ | - | +++ | +++ | - | +++ | ++ | ++ | ++ | - | ++ | ++ | ++ |
| S27 | +++ | - | +++ | +++ | +++ | + | - | ++ | ++ | +++ | +++ | - | - |
| S28 | +++ | - | +++ | +++ | - | +++ | - | ++ | ++ | - | ++ | ++ | ++ |
| S30 | + | - | +++ | +++ | ++ | +++ | - | - | ++ | - | ++ | - | ++ |
| S31 | + | - | +++ | +++ | ++ | +++ | - | - | ++ | - | ++ | + | ++ |
| M6 | ++ | - | +++ | +++ | +++ | - | ++ | - | ++ | ++ | ++ | - | - |
| M40 | ++ | - | + | ++ | ++ | - | - | ++ | ++ | ++ | ++ | - | - |
| Y37 | ++ | - | +++ | +++ | +++ | - | + | +++ | + | +++ | +++ | - | - |
| Y51 | + | - | +++ | +++ | +++ | - | - | +++ | ++ | ++ | ++ | - | - |
| Y52 | + | - | +++ | +++ | +++ | - | - | +++ | + | +++ | + | - | + |
| Y53 | ++ | + | +++ | +++ | +++ | + | ++ | +++ | ++ | +++ | +++ | + | + |
| Y45 | - | - | +++ | +++ | +++ | + | - | +++ | ++ | ++ | - | - | - |
| Y54 | ++ | - | +++ | +++ | ++ | - | - | +++ | ++ | +++ | + | - | - |
| Y56 | + | - | +++ | +++ | +++ | + | ++ | ++ | + | +++ | ++ | + | + |
| YC11 | - | - | +++ | +++ | - | - | - | ++ | ++ | +++ | +++ | - | - |

**Table S1. Estimation of the activities of secretory enzymes and the acid-producing abilities of *B. coagulans* species using different sugars.**

D/d＞2.5, ++++; 2<D/d≤2.5, +++; 1.5<D/d≤2, ++; 1<D/d≤1.5, +; D/d≤1, －. D: diameter of ydrolysis circle or staining circle (cm); d: colony diameter (cm).

**Table S2. Box-Behnken experimental design.**

| Code | Variable | Coding level | | |
| --- | --- | --- | --- | --- |
|  |  | -1 | 0 | 1 |
| A | Substrate-to-water | 0.75 | 1.0 | 1.25 |
| B | Inoculation amount (%v/w) | 10 | 15 | 20 |
| C | Fermentation time (h) | 36 | 48 | 60 |
| D | Expanded corn flour content (%) | 1 | 2 | 3 |
| E | FeSO_4_ content (%) | 0.6 | 0.8 | 1.0 |

**Table S3. ANOVA results of the quadratic model for the concentration of viable cells.**

| Y_1_ (concentration of viable cells) | | | | | |
| --- | --- | --- | --- | --- | --- |
| Source | Sum of Squares | df | Mean Square | *F*-Value | *P*-value |
| Model | 96.42 | 20 | 4.82 | 3.480 | 0.0028 |
| A | 0.28 | 1 | 0.28 | 0.203 | 0.6571 |
| B | 0.37 | 1 | 0.37 | 0.268 | 0.6096 |
| C | 46.51 | 1 | 46.51 | 33.540 | < 0.0001 |
| D | 1.36 | 1 | 1.36 | 0.983 | 0.3323 |
| E | 0.35 | 1 | 0.35 | 0.253 | 0.6199 |
| AB | 0.65 | 1 | 0.65 | 0.467 | 0.5014 |
| AC | 0.01 | 1 | 0.01 | 0.005 | 0.9465 |
| AD | 5.98 | 1 | 5.98 | 4.310 | 0.0498 |
| AE | 0.03 | 1 | 0.03 | 0.023 | 0.8799 |
| BC | 0.18 | 1 | 0.18 | 0.127 | 0.7248 |
| BD | 1.51 | 1 | 1.51 | 1.090 | 0.3076 |
| BE | 0.62 | 1 | 0.62 | 0.444 | 0.5120 |
| CD | 1.03 | 1 | 1.03 | 0.743 | 0.3980 |
| CE | 1.29 | 1 | 1.29 | 0.929 | 0.3456 |
| DE | 2.58 | 1 | 2.58 | 1.860 | 0.1867 |
| A² | 13.00 | 1 | 13.00 | 9.380 | 0.0057 |
| B² | 16.26 | 1 | 16.26 | 11.720 | 0.0024 |
| C² | 24.89 | 1 | 24.89 | 17.950 | 0.0003 |
| D² | 21.66 | 1 | 21.66 | 15.620 | 0.0007 |
| E² | 15.01 | 1 | 15.01 | 10.820 | 0.0033 |
| Residual | 30.51 | 22 | 1.39 |  |  |
| Lack of Fit | 30.30 | 20 | 1.51 | 14.330 | 0.0672 |
| R^2^ | 0.760 |  |  |  |  |
| Adequate Precision = 7.675, Standard Deviation =1.18, C.V.%=29.65 | | | | | |

**Table S4. ANOVA results of the quadratic model for the FG detoxification rate in solid-state fermentation of CSM.**

| Y_2_ (the detoxification efficiency of CSM) | | | | | | |  |
| --- | --- | --- | --- | --- | --- | --- | --- |
| Source | Sum of Squares | df | Mean Square | | *F*-Value | *P*-value | |
| Model | 336.08 | 20 | 16.80 | | 6.440 | < 0.0001 | |
| A | 0.73 | 1 | | 0.73 | 0.282 | 0.6010 | |
| B | 0.05 | 1 | | 0.05 | 0.020 | 0.8877 | |
| C | 1.01 | 1 | | 1.01 | 0.388 | 0.5400 | |
| D | 0.12 | 1 | | 0.12 | 0.046 | 0.8323 | |
| E | 254.50 | 1 | | 254.50 | 97.580 | < 0.0001 | |
| AB | 1.26 | 1 | | 1.26 | 0.482 | 0.4950 | |
| AC | 0.79 | 1 | | 0.79 | 0.304 | 0.5871 | |
| AD | 5.02 | 1 | | 5.02 | 1.930 | 0.1791 | |
| AE | 1.49 | 1 | | 1.49 | 0.570 | 0.4582 | |
| BC | 3.91 | 1 | | 3.91 | 1.500 | 0.2337 | |
| BD | 0.03 | 1 | | 0.03 | 0.010 | 0.9196 | |
| BE | 4.31 | 1 | | 4.31 | 1.650 | 0.2119 | |
| CD | 1.41 | 1 | | 1.41 | 0.540 | 0.4702 | |
| CE | 0 | 1 | | 0 | 0 | 1 | |
| DE | 2.50 | 1 | | 2.50 | 0.960 | 0.3379 | |
| A² | 33.02 | 1 | | 33.02 | 12.660 | 0.0018 | |
| B² | 1.35 | 1 | | 1.35 | 0.516 | 0.4800 | |
| C² | 33.18 | 1 | | 33.18 | 12.720 | 0.0017 | |
| D² | 4.98 | 1 | | 4.98 | 1.910 | 0.1811 | |
| E² | 19.96 | 1 | | 19.96 | 7.650 | 0.0113 | |
| Residual | 57.38 | 22 | | 2.61 |  |  | |
| Lack of Fit | 57 | 20 | | 2.85 | 15.250 | 0.0633 | |
| R² | 0.854 |  | |  |  |  | |
| Adequate Precision =10.273, Standard Deviation =1.61, C.V.%=1.91 | | | | | | |  |
